# An inventory of supranational antimicrobial resistance surveillance networks involving low- and middle-income countries since 2000

**DOI:** 10.1093/jac/dky026

**Published:** 2018-03-05

**Authors:** Elizabeth A Ashley, Judith Recht, Arlene Chua, David Dance, Mehul Dhorda, Nigel V Thomas, Nisha Ranganathan, Paul Turner, Philippe J Guerin, Nicholas J White, Nicholas P Day

**Affiliations:** 1 Myanmar-Oxford Clinical Research Unit (MOCRU), Yangon, Myanmar; 2 Centre for Tropical Medicine and Global Health, University of Oxford, Oxford, UK; 3 Mahidol-Oxford Tropical Medicine Research Unit (MORU), Faculty of Tropical Medicine, Mahidol University, Bangkok, Thailand; 4 Institute of Infectious Diseases and Epidemiology, Tan Tock Seng Hospital, Singapore; 5 Lao-Oxford-Mahosot Hospital-Wellcome Trust Research Unit (LOMWRU), Vientiane, Laos; 6 Faculty of Infectious and Tropical Diseases, London School of Hygiene and Tropical Medicine, London, UK; 7 WorldWide Antimicrobial Resistance Network (WWARN), Centre for Tropical Medicine and Global Health, University of Oxford, Oxford, UK; 8 Imperial College, London, UK; 9 Cambodia-Oxford Medical Research Unit (COMRU), Angkor Hospital for Children, Siem Reap, Cambodia; 10 Infectious Diseases Data Observatory (IDDO), Centre for Tropical Medicine and Global Health, University of Oxford, Oxford, UK

## Abstract

Low- and middle-income countries (LMICs) shoulder the bulk of the global burden of infectious diseases and drug resistance. We searched for supranational networks performing antimicrobial resistance (AMR) surveillance in LMICs and assessed their organization, methodology, impacts and challenges. Since 2000, 72 supranational networks for AMR surveillance in bacteria, fungi, HIV, TB and malaria have been created that have involved LMICs, of which 34 are ongoing. The median (range) duration of the networks was 6 years (1–70) and the number of LMICs included was 8 (1–67). Networks were categorized as WHO/governmental (*n *=* *26), academic (*n *=* *24) or pharma initiated (*n* = 22). Funding sources varied, with 30 networks receiving public or WHO funding, 25 corporate, 13 trust or foundation, and 4 funded from more than one source. The leading global programmes for drug resistance surveillance in TB, malaria and HIV gather data in LMICs through periodic active surveillance efforts or combined active and passive approaches. The biggest challenges faced by these networks has been achieving high coverage across LMICs and complying with the recommended frequency of reporting. Obtaining high quality, representative surveillance data in LMICs is challenging. Antibiotic resistance surveillance requires a level of laboratory infrastructure and training that is not widely available in LMICs. The nascent Global Antimicrobial Resistance Surveillance System (GLASS) aims to build up passive surveillance in all member states. Past experience suggests complementary active approaches may be needed in many LMICs if representative, clinically relevant, meaningful data are to be obtained. Maintaining an up-to-date registry of networks would promote a more coordinated approach to surveillance.

## Introduction

The burden of drug-resistant infections is increasing year on year. It has been predicted that the largest numbers of lives that will be lost as a result of these infections will be in low- and middle-income countries (LMICs).^1^ A global action plan on antimicrobial resistance (AMR) was endorsed in May 2015 by the World Health Assembly and calls upon countries to strengthen AMR surveillance. It is generally accepted that we need good AMR surveillance data to be able to assess the scale of the problem accurately and to guide interventions. Many LMICs are already participating in surveillance initiatives for AMR in malaria, TB, HIV and influenza. Attempts to kick-start global surveillance for resistance to commonly used antibacterial drugs have been made in the past but generally without success. The Global Antimicrobial Resistance Surveillance System (GLASS) was launched in 2015 with the goal of collecting comparable AMR data at country level for key bacterial pathogens.^2^ At the same time, the recent catastrophic Ebola epidemic in West Africa has brought the need for surveillance for emerging or epidemic-prone diseases into sharp focus, as experience has shown the majority of these have their origins in LMICs. The interaction between different drivers in humans, animals and the environment argues for adopting a ‘One Health’ approach to surveillance for both AMR and emerging diseases.

Here, we summarize the supranational surveillance networks for drug-resistant infections operating in LMICs since 2000 and discuss their impacts and challenges, and any implications for the implementation of GLASS.

## Methods

For the purposes of this analysis, AMR was defined as resistance to antimicrobial agents in bacteria, protozoa, fungi and viruses. Countries were categorized into income groups using the World Bank 2015 classification.^3^

### Search strategy

We searched for supranational networks performing AMR surveillance in LMICs from January 2000 to August 2017 in Embase, PubMed and Global Health databases. The search was performed first in May 2016 and updated in August 2017. Search terms were broad and included multiple alternative terms for AMR (e.g. drug resistance, antibiotic resistance, antifungal resistance, antimalarial drug resistance, antiviral resistance, cross resistance, multidrug resistance), as well as alternative terms for surveillance and for LMICs, which were also searched for individually by name (the complete list of search terms is available as Supplementary data at *JAC* Online). The titles and abstracts or full text of 20 558 (16 629 in 2016 plus 3929 in 2017) articles were screened to identify networks.

Networks did not have to collect primary samples to be included, i.e. they could collate resistance data collected by other groups. We excluded networks that occasionally reported drug resistance but did not have AMR surveillance as the major focus of their activity, e.g. a global travel-associated infection surveillance network, several One-Health networks and the Digital Disease Detection networks (e.g. ProMed). Networks were categorized by type (WHO/governmental, academic, pharmaceutical company/contract research organization-led or other), target pathogen grouping (bacteria, TB, malaria, HIV, other) and funding source. Networks performing AMR surveillance in bacteria were further characterized by pathogen sub-group (e.g. respiratory, enteric) and population under surveillance (e.g. community versus hospital-acquired infection, children). We noted the approaches to quality management taken and impacts or challenges of the networks when recorded.

## Results

**Figure 1.**
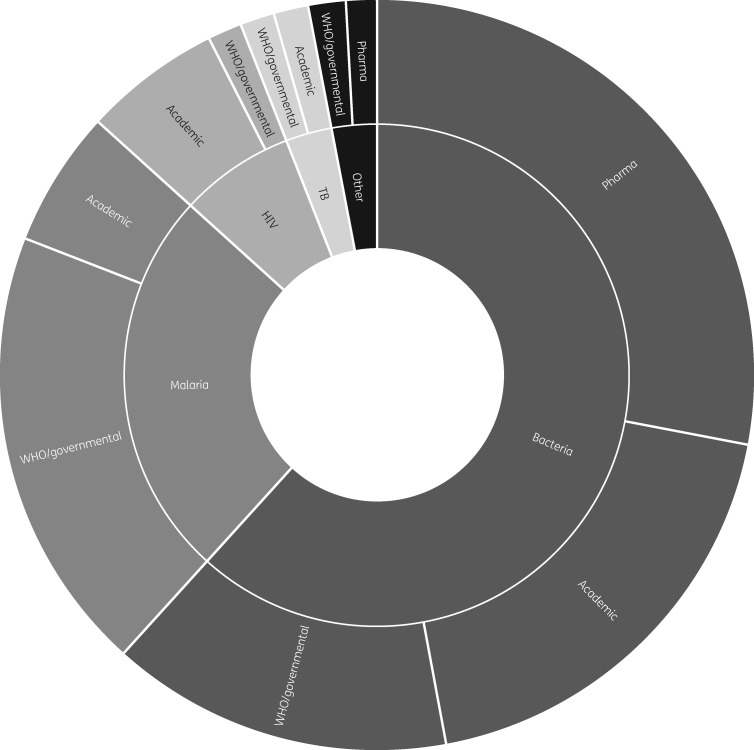
Sunburst chart of network types.

We identified 72 supranational networks concerned with AMR surveillance since 2000, of which 26 were WHO/governmental (global or regional), 24 academic and 22 pharma initiated (Figure 1). Funding sources varied, with 30 networks receiving public or WHO funding, 25 corporate, 13 trust or foundation, and 4 funded from more than one type of source. The data-sharing models of the networks were open access (*n *=* *3), closed (*n *=* *38) and shared or unclear (*n *=* *31).

In terms of the pathogens under surveillance, 45 networks were for AMR in bacteria or fungi (Table 1), 18 in malaria, 2 in TB, 6 in HIV and 1 for influenza (Table 2). The median (range) duration of the networks was 6 years (1–70). In the case of the discontinued malaria networks, inability to secure sustainable funding was an important reason for their collapse.^4^ Coverage of LMICs by the networks varied greatly. The median (range) number of LMICs included in the AMR surveillance networks for which the information was available was 8 (1–67). The WHO Global Influenza Surveillance and Response System (WHO GISRS) was the longest running network, established in 1947, and included the greatest number of LMICs (67), although antiviral resistance was not under surveillance at the outset.

**Table 1.** AMR surveillance networks for bacteria and fungi in LMICs (arranged in alphabetical order)

	Name (acronym), coordinating institution (if different)	Pathogen category, network type, funding type	No. of LMICs/no. of countries	Years active	Description
1	The Alexander Project, GlaxoSmithKline	bacteria	4/32	1992–2002	longitudinal multicentre surveillance of antimicrobial susceptibility of community-acquired respiratory pathogens
		pharma/CRO			
		corporate			
2	Asian Network for Surveillance of Resistant Pathogens (ANSORP), Sungkyunkwan University, Korea	bacteria	8/14	1996–ongoing	academic regional network with varied research portfolio; funding sought for individual projects
		academic			
		corporate, public, trust or foundation			
3	Antimicrobial Resistance Epidemiological Survey on Cystitis (ARESC), European Society for Infection in Urology	bacteria	1/10	2003–06	survey of women symptomatic of urinary tract infection (predominantly in Europe)
		academic			
		corporate			
4	Antibiotic Resistance in the Mediterranean Region (ARMed), Infection Control Unit, Mater Dei Hospital, Msida, Malta	bacteria	7/9	2003–07	multicentre hospital-based study of AMR, antibiotic use and infection control practices
		WHO/governmental			
		public			
5	ARTEMIS Global Antifungal Surveillance Programme (ARTEMIS)	fungi	9/34	1997–2005	longitudinal multicentre surveillance of *Candida* spp. and non-candidal yeasts
		pharma/CRO			
		corporate			
6	Assessing Worldwide Antimicrobial Resistance and Evaluation Programme (AWARE), International Health Management Associates, Inc. (IHMA)	bacteria	3/7	2012–ongoing	ceftaroline surveillance programme
		pharma/CRO			
		corporate			
7	Bacterial Infections and Antibiotic-Resistant Diseases Among Young Children in Low-Income Countries (BIRDY), Institut Pasteur International Network	bacteria	3/3	2012–ongoing	multinational, longitudinal cohort study of community-acquired and nosocomial infections and drug resistance in children
		academic			
		corporate, public, trust or foundation			
8	Central Asian and Eastern European Surveillance of Antimicrobial Resistance (CAESAR)	bacteria	17/20	2013–ongoing	European AMR surveillance network for non-EU countries
		WHO/governmental			
		public			
9	Caribbean Public Health Agency (CARPHA)	bacteria	10/25	2013–ongoing	AMR surveillance is one of the agency’s core activities
		WHO/governmental			
		trust or foundation			
10	Community-Acquired Respiratory Tract Infection Pathogen Surveillance (CARTIPS)	bacteria	2/4	2009–10	Asian multicentre AMR surveillance of community-acquired respiratory pathogens
		pharma/CRO			
		corporate			
11	Centre for Disease Dynamics, Economics and Policy (CDDEP)/ResistanceMap	bacteria	NS	1999–ongoing	ResistanceMap uses interactive maps and charts to visualize AMR (and antimicrobial use) data
		academic			
		trust or foundation, public			
12	Community-Based Surveillance of Antimicrobial Use and Resistance in Resource-Constrained Settings, WHO	bacteria	2/2	2002–05	pilot AMR and AMU surveillance projects at five sites in India and South Africa
		academic			
		public			
13	Comparative Activity of Carbapenem Testing (COMPACT and COMPACT II), Janssen Asia-Pacific	bacteria	3/5	2008–10	assessment of carbapenem susceptibility of Gram-negative bacteria isolated from hospitalized patients in the Asia-Pacific region
		pharma/CRO			
		corporate			
14	International Daptomycin Surveillance Programmes, JMI Laboratories	bacteria	12/33	2011–ongoing	assessment of daptomycin susceptibility of various Gram-positive clinical isolates
		pharma/CRO			
		corporate			
15	Diseases of the Most Impoverished Typhoid Study Group and Multicentre Shigellosis Surveillance Study (DOMI), International Vaccine Institute, Republic of Korea	bacteria	5/5	2001–04	population-based surveillance studies in Asia with antimicrobial susceptibility of isolates from confirmed cases
		academic			
		trust or foundation			
16	European Antimicrobial Resistance Surveillance Network (EARS-Net), ECDC	bacteria	2/29	1999–ongoing	European AMR surveillance network for EU countries
		WHO/governmental			
		public			
17	Enter-Net International Surveillance Network, Health Protection Agency, UK	bacteria	1/28	1993–2007	European foodborne infection/AMR surveillance network; transferred to ECDC (FWD-Net)
		WHO/governmental			
		public			
18	Food- and Waterborne Diseases and Zoonoses Network (FWD-Net), ECDC	bacteria	2/29	2007-ongoing	European surveillance network for food- and waterborne diseases (includes AMR), for EU countries
		WHO/governmental			
		public			
19	Gonococcal Antimicrobial Surveillance Programme (GASP), WHO	bacteria	32/70	1992–ongoing	global network for sentinel surveillance of AMR (especially cephalosporins) in *N. gonorrhoeae*
		WHO/governmental			
		public			
20	Global Point Prevalence Survey of Antimicrobial Consumption and Resistance (Global-PPS), University of Antwerp	bacteria	24/63	2015–ongoing	multicentre point prevalence survey of antimicrobial prescribing and resistance in hospitalized patients
		academic			
		corporate			
21	International Network For Optimal Resistance Monitoring (INFORM), IHMA	bacteria	NS	2012–14	Asia-Pacific, Latin America, Middle East, Africa, Europe
		pharma/CRO			
		corporate			
22	International Nosocomial Infection Control Consortium (INICC)	bacteria	32/43	2002–ongoing	main focus is on the reduction of healthcare-associated infections; collects associated AMR data
		academic			
		trust or foundation			
23	International Network for the Study and Prevention of Emerging Antimicrobial Resistance (INSPEAR), US CDC	bacteria	9/30	1998–2010	AMR early warning system with proficiency testing for laboratories and expedited reporting of drug-resistant infections
		academic			
		public			
24	In Vitro Activity of Oral Antimicrobial Agents Against Pathogens Associated With Community-Acquired Upper Respiratory Tract and Urinary Tract Infections: A Five Country Surveillance Study, IHMA	bacteria	2/5	2012–13	global surveillance of susceptibility of community-acquired respiratory and urinary tract pathogens
		pharma/CRO			
		corporate			
25	Multiyear, Multinational Survey of the Incidence and Global Distribution of MBL-Producing Enterobacteriaceae and *Pseudomonas aeruginosa*, IHMA	bacteria	∼12/31	2012–14	global hospital-based surveillance of MBL-producing Gram-negative bacteria
		pharma/CRO			
		corporate			
26	Minocycline activity tested against *Acinetobacter baumannii* complex, *Stenotrophomonas maltophilia* and *Burkholderia cepacia* species complex isolates from a global surveillance programme (2013), JMI Laboratories	bacteria	NS/46	2013	AMR surveillance in Gram-negative organisms focused on assessment of minocycline activity
		pharma/CRO			
		corporate			
27	Meropenem Yearly Susceptibility Test Information Collection (MYSTIC), AstraZeneca	bacteria	8/21	1997–2008	assessment of meropenem susceptibility of various clinical isolates from patients with serious infections.
		pharma/CRO			
		corporate			
28	Network for Surveillance of Pneumococcal Disease in the East Africa Region (netSPEAR)	bacteria	4/4	2003–07	East African network that strengthened routine surveillance of *Streptococcus pneumoniae* and *Haemophilus influenzae* infections in children (laboratory and data-management training, improved communication)
		academic			
		trust or foundation			
29	NosoMed Pilot Survey in the Eastern Mediterranean Area, Université Claude Bernard Lyon I	bacteria	2/3	2003–04	multicentre surveillance of drug-resistant nosocomial bacterial isolates
		academic			
		public			
30	Programme to Assess Ceftolozane/Tazobactam Susceptibility (PACTS), Cubist Pharmaceuticals	bacteria	2/16	2012–ongoing	ceftolozane/tazobactam susceptibility surveillance programme focused on nosocomial infections
		pharma/CRO			
		corporate			
31	Pan-European Antimicrobial Resistance Using Local Surveillance (PEARLS), Wyeth Pharmaceuticals	bacteria	4/17	2001–02	AMR surveillance of nosocomial isolates of *Enterococcus faecium, Enterobacter cloacae, Enterobacter aerogenes, E. coli, Klebsiella pneumoniae, S. aureus*
		pharma/CRO			
		corporate			
32	Prospective Resistant Organism Tracking and Epidemiology for the Ketolide Telithromycin (PROTEKT), Sanofi-Aventis	bacteria	10/36	1999–2004	international AMR surveillance of community-acquired respiratory tract pathogens
		pharma/CRO			
		corporate			
33	Red Latinoamericana de Vigilancia de la Resistencia a los Antimicrobianos (ReLAVRA), PAHO	bacteria	15/19	1996–ongoing	Latin-American AMR surveillance network with a proficiency testing programme
		WHO/governmental			
		public			
34	South Asian Pneumococcal Alliance (SAPNA), GAVI Alliance	bacteria	3/3	2004–09	AMR surveillance of infections caused by *S. pneumoniae, H. influenzae* and *N. meningitidis* in South Asian children
		academic			
		public, corporate			
35	Study on Antimicrobial Resistance in *Staphylococcus aureus* (SARISA), LEO Pharma (Copenhagen)	bacteria	2/18	1996–ongoing	multicentre survey of AMR in *S. aureus*
		pharma/CRO			
		corporate			
36	SENTRY Antimicrobial Surveillance Programme, JMI laboratories	bacteria, fungi	∼8/40	1997–ongoing	monitors antimicrobial susceptibility in a wide variety of community-acquired and nosocomial pathogens
		pharma/CRO			
		corporate			
37	Sistema de Redes de Vigilancia de los Agentes Responsables de Neumonias y Meningitis Bacterianas (SIREVA and SIREVA II), PAHO	bacteria	15/19	1993–ongoing	Latin-American regional network for surveillance of respiratory and meningitis pathogens
		WHO/governmental			
		public			
38	Study for Monitoring Antimicrobial Resistance Trends (SMART), Merck & Co. Inc.	bacteria	23/53	2002–11	AMR surveillance of Gram-negative clinical isolates from intra-abdominal infections and urinary tract infections
		pharma/CRO			
		corporate			
39	Survey of Antibiotic Resistance (SOAR), GlaxoSmithKline	bacteria	34/48	2002–ongoing	a series of studies of antimicrobial susceptibility of pathogens causing community-acquired respiratory infection
		pharma/CRO			
		corporate			
40	International Solithromycin Surveillance Programmes, JMI Laboratories, USA	bacteria	5/27	2011–ongoing	AMR surveillance in Gram-positive organisms focused on assessment of solithromycin activity
		pharma/CRO			
		corporate			
41	TARGETed Surveillance Study, GR Micro Ltd, UK	bacteria	2/7	2003–07	AMR surveillance of community-acquired respiratory tract pathogens with a focus on fluoroquinolone activity
		pharma/CRO			
		corporate			
42	Tigecycline Evaluation and Surveillance Trial (TEST), IHMA	bacteria	25/65	2004–ongoing	global, hospital-based AMR surveillance of a wide range of clinical isolates with a focus on tigecycline susceptibility
		pharma/CRO			
		corporate			
43	Typhoid Fever Surveillance in Africa Programme (TSAP), International Vaccine Institute, Korea	bacteria	10/10	2009–ongoing	multinational, population surveillance study of typhoid incidence in Africa (included AST of invasive isolates)
		academic			
		trust or foundation			
44	WHO Western Pacific Regional Programme for Surveillance of Antimicrobial Resistance	bacteria	6/13	1991–98	regional network for antimalarial therapeutic efficacy monitoring
		WHO/governmental, academic			
		public			
45	Zyvox^®^ Annual Appraisal of Potency and Spectrum (ZAAPS), JMI Laboratories, USA and Pfizer	bacteria	12/42	2004–ongoing	global monitoring of linezolid activity against Gram-positive bacteria
		pharma/CRO			
		corporate			

PAHO, Pan American Health Organization; CRO, contract research organization; NS, not specified.

**Table 2.** AMR surveillance networks for malaria, HIV, TB and influenza in LMICs (arranged by pathogen and in alphabetical order)

	Name (acronym), coordinating institution if different	Pathogen category, network type, funding type	No. of LMICs/no. of countries	Years active	Description
Malaria					
1	Amazon Malaria Initiative (AMI), PAHO	malaria	11/12	2001–ongoing	Latin-American regional antimalarial resistance surveillance network; some overlap with RAVREDA
		WHO/governmental			
		public			
2	Artemisinin Resistance Confirmation, Characterization and Containment Collaboration (ARC3), WHO	malaria	3/3	2009–10	multicentre study of artemisinin resistance in Southeast Asia
		academic			
		trust or foundation, public			
3	Artemisinin Resistance Containment and Elimination Collaboration (ARCE), WHO	malaria	3/3	2010–11	multicentre artemisinin-resistant malaria containment and elimination project
		academic			
		trust or foundation, public			
4	Bangladesh, Bhutan, India, Nepal, Sri Lanka Malaria Drug Resistance Network (BBINS)	malaria	5/5	2011–ongoing	regional network for antimalarial therapeutic efficacy monitoring
		WHO/governmental			
		public			
5	East African Network for Monitoring Antimalarial Treatment (EANMAT)	malaria	5/5	1997–2006	regional network for antimalarial therapeutic efficacy monitoring
		WHO/governmental, academic			
		public			
6	Greater Mekong Sub-region Therapeutic Efficacy Studies (TES) Network	malaria	8/8	2007–ongoing	regional network for antimalarial therapeutic efficacy monitoring
		WHO/governmental			
		public			
7	Horn of Africa Network for Monitoring Antimalarial Treatment (HANMAT)	malaria	5/6	2004–ongoing	regional network for antimalarial therapeutic efficacy monitoring
		WHO/governmental			
		public			
8	K13 Artemisinin Resistance Multicentre Assessment Consortium (KARMA), Institut Pasteur	malaria	56/59	2014–ongoing	multinational molecular genotyping trials to map the kelch 13 mutation
		academic			
		public			
9	MalariaGEN Genomic Epidemiology Network, MalariaGEN Resource Centre	malaria academic trust or foundation	∼36/36	2005–ongoing	Global network focusing on analysis of genetic/genomic data
10	Plasmodium Diversity Network Africa (PDNA), University of Science, Techniques and Technologies, Bamako, Mali	malaria	15/15	2012–ongoing	African network mapping malaria parasite genetic diversity and molecular markers of drug resistance
		academic			
		public, trust or foundation			
11	Pacific Malaria Drug Resistance Monitoring Network	malaria	7/8	2011–ongoing	regional network for antimalarial therapeutic efficacy monitoring
		WHO/governmental			
		public			
12	Pakistan-Iran-Afghanistan Malaria Network	malaria	3/3	2008–ongoing	regional network for antimalarial therapeutic efficacy monitoring
		WHO/governmental			
		public			
13	Reseau d'Afrique Centrale pour Traitement Anti-Paludisme (RACTAP)	malaria	8/8	2003–09	regional network for antimalarial therapeutic efficacy monitoring
		WHO/governmental			
		public			
14	Amazon Network for the Surveillance of Antimalarial Drug Resistance (RAVREDA)	malaria	12/13	2001–ongoing	regional network for antimalarial therapeutic efficacy monitoring
		WHO/governmental			
		public			
15	South African Network for the Monitoring of Antimalarial Drug Resistance (SANMAT)	malaria	7/7	2002–14	regional network for antimalarial therapeutic efficacy monitoring
		WHO/governmental academic			
		public			
16	Tracking Resistance to Artemisinin Collaboration (TRAC and TRAC2), Mahidol Oxford Tropical Medicine Research Unit	malaria	10/10	2011–ongoing	multinational clinical trials to map artemisinin resistance
		academic			
		public			
17	West African Network for Monitoring Antimalarial Treatment (WANMAT)	malaria	15/15	2003–09	regional network for antimalarial therapeutic efficacy monitoring
		WHO/governmental, academic			
		public			
18	WorldWide Antimalarial Resistance Network (WWARN)	malaria	37/70	2009–ongoing	collates antimalarial resistance data from other groups and performs individual patient data meta-analyses
		academic			
		corporate, trust or foundation			
HIV					
1	Europe Africa Research Network for Evaluation of Second-Line Therapy (EARNEST)	HIV	4/4	2010–11	academic network focused on HIV resistance to second-line therapies in Africa
		academic			
		public			
2	Global HIV Drug Resistance Network (HIVResNet), WHO	HIV	15/23	2007–ongoing	global network of experts from academic institutions, laboratories and international and non-profit organizations
		WHO/governmental			
		public			
3	International Epidemiologic Databases to Evaluate AIDS (IeDEA), NIAID	HIV	36/47	2005–ongoing	platform for data sharing from different sites, used to address research questions
		academic			
		public			
4	PharmAccess African Studies to Evaluate Resistance (PASER), PharmAccess Foundation, AIGHD and Virology Department at the University Medical Centre, Utrecht, The Netherlands	HIV	6/6	2006–15	multinational HIV DR surveillance in Africa
		academic			
		public			
5	TREAT Asia Studies to Evaluate Resistance (TASER)	HIV	5/6	2007–11	HIV DR surveillance programme linked to TREAT Asia studies
		academic			
		public, trust or foundation			
6	Tenofovir Resistance Study Group (TenoRES)	HIV	10/23	2015–16	pooled-data analysis of tenofovir and other antiretroviral resistance in HIV
		academic			
		trust or foundation			
TB					
1	Comprehensive Resistance Prediction for Tuberculosis International Consortium (CRyPTIC), University of Oxford	TB	5/10	2015–ongoing	WGS of isolates from multiple locations to investigate genomic variation associated with drug resistance
		academic			
		trust or foundation			
2	WHO/IUATLD Global Project on Anti-Tuberculosis Drug Resistance Surveillance (WHO/IUATLD)	TB	39/89	1994–ongoing	global surveillance programme with associated supranational reference laboratory network
		WHO/governmental			
		public			
Influenza					
1	WHO Global Influenza Surveillance and Response System (WHO GISRS)	influenza	67/113	1947–ongoing	global surveillance for susceptibility of influenza viruses to neuraminidase inhibitors
		WHO/governmental			
		public			

AIGHD, Amsterdam Institute for Global Health and Development; PAHO, Pan American Health Organization; DR, drug resistance.

### Networks for AMR surveillance in bacterial pathogens

Of the 44 networks focused on AMR in bacteria, 6 reported data on the GLASS priority pathogens (with the exception of *Salmonella* spp. in 4), 2 networks were for *Staphylococcus aureus*, 10 were for respiratory pathogens (2 of these included *Neisseria meningitidis* and 1 enteric pathogens), 4 were for enteric pathogens only, 1 was for *Neisseria gonorrhoeae* and the remainder included a range of Gram-negative (5) or Gram-positive (2) bacteria or a mixture of the two. Seven networks collected or reported data on invasive isolates only, five non-invasive only and the remainder both. For those networks that specified the patient populations isolates came from, seven were community-acquired, five hospital-acquired, one was in women and four in children.

### Differences between network categories

The networks were a heterogeneous group with different approaches to surveillance reflecting different objectives. The greatest diversity was found in the antibacterial surveillance group. Most global networks initiated and sponsored by pharmaceutical companies had the objective of evaluating susceptibility to specific drugs (registered drugs or new compounds). A variety of bacterial or fungal pathogens were collected by the pharma networks including community- and hospital-acquired isolates from both sterile and non-sterile sites. Academic networks tended to focus AMR surveillance around a specific clinical question, e.g. one project of the Asian Network for Surveillance of Resistant Pathogens (ANSORP) evaluated susceptibility of ESBL-producing isolates collected in the region to different antimicrobials (Tables 1 and 2). Other academic networks such as the WorldWide Antimalarial Resistance Network (WWARN) part of the newly established Infectious Diseases Data Observatory (IDDO) and International Epidemiologic Databases to Evaluate AIDS (IeDEA) have led analyses of individual patient data collected by other research groups.

The approaches taken for drug resistance surveillance by the major global programmes (TB, malaria, HIV, bacteria, influenza) are summarized in Table 3. As shown, the TB, malaria and HIV networks take an active approach to AMR surveillance in LMICs while the antibacterial and influenza networks rely on case-based surveillance from sentinel sites.

**Table 3.** Approaches to AMR surveillance taken by global WHO programmes in LMICs

	TB	Malaria	HIV	Bacteria (GLASS + GASP)	Influenza (GISRS)
Type(s) of surveillance	epidemiological surveys or case notification	therapeutic efficacy studies at sentinel sites and molecular marker surveys	EWI^a^; two types of molecular marker surveys (PDR and ADR)^b^	routine surveillance of clinical isolates at sentinel sites	case-based surveillance from sentinel sites
Technology/laboratory methods	culture and susceptibility testing; GeneXpert^®^; other molecular methods	microscopy and PCR-based technologies	PCR based	culture and susceptibility testing	RT-PCR based; HAI test; virus isolation in cell culture and susceptibility testing at reference laboratories
Defined selection criteria for population of interest	yes	yes	yes	no	yes – clinical case definition
Recommended sample size	yes	yes	yes	no	no
Recommended frequency of surveillance	every 5 years (survey-based methodology); continuous (if case-based surveillance)	every 3 years	every 3 years	annual	continuous
Data sharing mechanism	WHO Global TB Database	WHO Global Malaria Programme Database	no	WHONET	FluNet
Regional surveillance networks	no	yes	no	yes	no
	surveillance data consolidated in WHO regional offices	BBINS, MBDS network, HANMAT, RAVREDA, PDRMN (other regional networks have collapsed due to lack of funding)	HIVResNet is a global network of experts from academic institutions, laboratories and non-profit organizations created in 2007 to develop strategies to monitor HIV DR	GLASS: Europe (EARS-Net; CAESAR) and Latin America (ReLAVRA); GASP data collated via WHO Regional Office/Reference Centres (except Africa)	GISRS is a network of National Influenza Centres (NICs) and WHO Collaborating Centres (WHOCCs)
Reference laboratory network(s)	yes	no	yes	–	yes
	WHO TB supranational reference laboratory network		global HIV drug resistance laboratory network	GLASS—no; GASP—yes	NICs; WHOCCs; WHO H5 Reference Laboratories
Global proficiency testing scheme	yes	no	no	no	yes
			participation in an EQA scheme is a prerequisite to becoming a WHO-designated genotyping laboratory	no global scheme proposed in GLASS; ReLAVRA—LA-EQAS; CAESAR—UK-NEQAS; GASP - EQAS	WHO-EQAP
Guidance on use of AMR surveillance data	individual case management and to guide design of new second-line treatment regimens	defined cut-offs for considering national treatment policy change	used to support choice of nationally recommended ART and prophylaxis regimens	to inform treatment guidelines	to improve antiviral use in treatment and for pandemic preparedness
Frequency of reporting	annual	every 5 years^20^	ad hoc; HIV DR global action plan under development	GLASS—first report January 2018; GASP—ad hoc (every 3 years approx.)	biennial (influenza virus surveillance reporting is available in real time)

MBDS, Mekong Basin Disease Surveillance; PDRMN, Pacific Malaria Drug Resistance Monitoring Network; DR, drug resistance.

a EWI = early warning indicator, e.g. antiretroviral coverage, retention in care, treatment interruption and viral load suppression.

b ADR = acquired HIV drug resistance and PDR = pretreatment HIV drug resistance.

### Networks for AMR surveillance in animals

There is one supranational European network for surveillance of food- and waterborne diseases and zoonoses that collects data on antimicrobial susceptibility in humans, animals and food. Larger networks that monitor foodborne infections [WHO Global Foodborne Infections network (GFN) and PulseNet International], including animal and environmental isolates, do not report AMR data although GFN does support an external quality assurance (EQA) programme for participating laboratories, which includes antimicrobial susceptibility testing (AST). No other supranational networks for AMR surveillance in animals were identified.

### Quality management

The networks had different approaches to quality management (Table 4). The pharma-led networks typically did not involve LMIC laboratories in EQA programmes but sent all isolates to a central laboratory for confirmatory testing. The global surveillance programmes for AMR in TB, HIV, influenza and gonorrhoea all had proficiency testing programmes delivered via supranational networks of reference laboratories. Among the networks for AMR surveillance in bacteria, the Latin-American network, Red Latinoamericana de Vigilancia de la Resistencia a los Antimicrobianos (ReLAVRA) has been running an EQA scheme (LA-EQAS) since 2000 and provides proficiency testing services at no cost to participating laboratories. The Central Asian and Eastern European Surveillance of Antimicrobial Resistance (CAESAR), the non-EU European network, has used the UK National External Quality Assessment Service (UK-NEQAS) for EQA. WHO-sponsored EQA efforts for AST included the discontinued WHO EQAS AST (1998–2001)^5^ and the WHO-AFRO/NICD-SA EQAP for countries within the WHO-AFRO region.^6^ Currently GLASS recommends national reference laboratories take responsibility for quality management.

**Table 4.** AMR-related proficiency testing and quality management in the networks

	Name (acronym) of programme/country location of head office	Years active	Description
1	Global Laboratory Initiative (GLI), for the Global TB Programme/Switzerland	2008–ongoing	standards and/or policy setting, proficiency testing, training
2	WHO HIVResNet Laboratory Accreditation Scheme/Switzerland	2007–ongoing	accreditation body; national HIV drug resistance working groups coordinating WHO-recommended surveys must use a WHO-designated genotyping laboratory
3	ReLAVRA Latin America External Quality Assessment (LA-EQAS)/Argentina	2000–ongoing	proficiency testing for the ReLAVRA network
4	TREAT Asia Quality Assessment Scheme(TAQAS)/Australia	2006–ongoing	TREAT Asia (an amfAR programme) aims to standardize HIV-1 genotypic resistance testing among laboratories to permit comparison of results from different centres
5	UK External Quality Assurance Scheme (UK NEQAS)/UK	1969–ongoing	offers proficiency testing in bacteriology and other laboratory disciplines; >8000 labs from over 140 countries participate
6	World Health Organization (WHO)/Switzerland	2003–ongoing	issues guidelines and sets policy
7	WHO African Region External Quality Assurance Programme (WHO AFRO EQAP)/South Africa	2002–ongoing	proficiency testing; 81 laboratories from 45 countries in the WHO African Region participate in the programme; in 2012 it was reported that 20% of labs did not respond to the surveys
8	WHO External Quality Assessment Project for the Detection of Subtype Influenza A Viruses by PCR/Switzerland	2007–ongoing	the EQA Project is conducted jointly by WHO Headquarters, WHO H5 Reference Laboratory and National Influenza Centre, China Hong Kong SAR, with support from the WHO Collaborating Centres on influenza and other WHO H5 Reference Laboratories
9	WHO Global Foodborne infections Network (WHO GFN) EQAS/Denmark	2000–ongoing	proficiency testing (pathogen identification, serotyping and AST) organized by the National Food Institute, Denmark
10	WHO Gonococcal Surveillance Programme EQAS	1992–ongoing	WHO Collaborating Centre in Sydney manages SE Asia/Asia-Pacific programmes
11	WHO Mycobacterial Supranational Reference Laboratory (SRL) Network/Switzerland	1991–ongoing	network of 33 laboratories providing reference laboratory services and proficiency testing
12	WHO External Quality Assurance System for Antimicrobial Susceptibility Testing (EQAS-AST)/Switzerland	1998–2006	proficiency testing programme in bacterial isolates (identification and AST)

### Impacts and challenges of the networks

Impacts and challenges of the networks were not recorded consistently. The main themes are summarized in Table 5 with examples. The biggest challenges faced by the global networks have been achieving high coverage across LMICs and complying with the recommended frequency of reporting. The Global Project on Anti-Tuberculosis Drug Resistance Surveillance has collected resistance data from 155/194 member states since its inception in 1994. For 72 countries without routine drug susceptibility testing of cases these data come from surveys, which are ideally performed every 5 years. The biggest gaps in surveillance in the most recent report were over West and central Africa. At an individual level it was estimated that 33% of new TB cases and 60% of cases treated previously underwent rifampicin susceptibility testing in 2016.^7^ Only one-third of 106 malaria endemic countries were in compliance with the recommended targets for antimalarial drug efficacy surveillance (monitoring at three-yearly intervals) when last reported, although the Global Malaria Programme has recently updated its web site with aggregate data from more studies.^8^^,^^9^ The Gonococcal Antimicrobial Surveillance Programme (GASP) has had no regional focal point in Africa since 2012. The WHO 2014 Global Report on Surveillance obtained data on antimicrobial susceptibility in *N. gonorrhoeae* from only 42/194 (22%) member states and noted that coverage was poorest from presumed high-burden countries. WHO GISRS reported resistance to the neuraminidase inhibitors of influenza viruses in 2016. Out of 13 312 viruses collected by National Influenza Centres between May 2014 and May 2015, 94% were from three WHO regions: Western Pacific, the Americas and Europe, with only 3% from Africa and 2% from Southeast Asia.^10^ WHO is in the process of developing a new Global Action Plan for HIV drug resistance. In July 2016 it was reported that 59/144 LMICs had monitored for the emergence of HIV drug resistance using the recommended early warning indicator system, which looks at antiretroviral treatment coverage, retention in care, treatment interruption and viral load suppression.^11^ A meta-analysis in 2012 reported HIV-1 drug resistance surveillance data from 42 LMICs between 2001 and 2011, and 8 countries performed surveys for pre-treatment HIV DR between 2014 and May 2016.^12^^,^^13^

**Table 5.** Impacts and challenges of the AMR surveillance networks in LMICs with examples

Impacts	Challenges
Led to changes in treatment policy (malaria networks) Improved laboratory capacity by establishing networks of reference laboratories and quality management systems (ARMed, WHO/IUATLD, GASP, ReLAVRA, CAESAR) Standardization of surveillance methodologies and data analysis (WHO Global Malaria Programme, ReLAVRA, WHO/IUATLD, HIVResNet, WHONET, WWARN) Reduction in healthcare-associated infections in countries (INICC)^21^^,^^22^ Exchange of information, training and knowledge between countries (WHO, ReLAVRA, WWARN, netSPEAR) Data sharing with secondary benefits to inform treatment guidelines (WWARN, IeDEA) Created global repositories of bacterial isolates; these can be used to screen new drugs (SENTRY, ANSORP) Discovery of new resistance mechanisms (The Alexander Project)	Low coverage, particularly in sub-Saharan Africa and India (GASP, GISRS) Lack of representativeness of data, e.g. due to selective sampling (HIV, GASP, some CAESAR sites) Difficulties of implementing routine blood culture/diagnostic microbiology in clinical practice (CAESAR) Difficulties in implementing complex surveillance methodologies, e.g. optimal *in vivo* methods for surveillance for artemisinin resistance in malaria, second-line drug susceptibility testing for TB Lack of engagement by some partners (netSPEAR) Reporting delays Sustainability due to underfunding with consequent understaffing; surveillance has generally not been given high priority by external donors (EANMAT, netSPEAR)

In a detailed account of the experience of setting up the Network for Surveillance of Pneumococcal Disease in the East Africa Region (netSPEAR), an East African network funded by the GAVI Alliance, in which routine surveillance for pneumococcal disease in public hospitals was strengthened, key challenges noted were difficulty in engaging the government of one of the participating countries in the network, poor performance of some sites despite training and problems with attracting funding.^14^ The importance of national and institutional ownership of surveillance activity and of framing it as part of routine activity rather than extra work was stressed. The benefits of collaboration between policymakers, academics and service providers were highlighted, a sentiment echoed by the experience of the malaria regional networks, which re-energized surveillance and also played a role in advocacy for policy change, acting as a bridge between research groups and national control programmes.^4^ Individual patient data meta-analyses coordinated by WWARN have led to policy recommendations to change antimalarial drug dosing. Another impact of the academic malaria drug efficacy surveillance networks has been the establishment of successful North–South scientific partnerships. There are a few examples where the scientific leadership now comes from the South, e.g. *Plasmodium* Diversity Network Africa, a molecular surveillance network.^15^

Surveillance networks have a positive impact by connecting laboratories in different countries. The Antibiotic Resistance in the Mediterranean Region (ARMed) network, which ran between 2003 and 2007, reported improvement in participating laboratories’ capacity to perform bacterial identification and AST, as a result of the EQA programme attached to the network.^16^ The HIV, mycobacteria, influenza and gonorrhoea reference laboratory networks have been created thanks to global surveillance programmes.

## Discussion

Defining the global burden of AMR and monitoring the impact of interventions to counter it requires reliable surveillance data. LMICs shoulder the bulk of the global burden of infectious diseases and drug resistance but their surveillance systems tend to be weaker than those in high-income countries (HICs), because passive surveillance cannot be integrated with routine case-management of patients easily in many areas. This problem has been circumvented to an extent in TB, malaria and HIV AMR surveillance by using active approaches to surveillance in LMICs and gathering data intermittently to provide a snapshot of the situation. However, achieving high coverage of all LMICs and complying with the recommended frequency of surveillance has been difficult. A review of the HIV, TB and malaria surveillance systems in 2011 suggested that one risk of integrating surveillance into routine activities was that high-quality implementation was less likely.^17^ By contrast, GLASS is based on building up or strengthening traditional models of passive case-based surveillance to generate data, as in HICs. Priority pathogens, drugs and specimens for surveillance are named but, unlike the other networks, GLASS does not specify minimum sample sizes or detailed selection criteria for target populations. Responsibility for quality management is devolved to national reference centres rather than a supranational body. Member states are requested to submit their AMR data to the WHO global antimicrobial susceptibility database (WHONET). The experiences of ReLAVRA, the Latin-American network, and, to an extent, CAESAR, the newer European network, have shown that case-based surveillance can be implemented in middle-income countries but obtaining representative data may take time. It is likely that it will be many more years before most low-income countries have a well-functioning system for routine bacteriological surveillance with high coverage. As a result, this approach risks generating non-representative data in the short- to medium- term, as has happened so far, and making inter-country comparisons will be difficult. The long-established WHO/International Union Against Tuberculosis and Lung Disease (WHO/IUATLD) surveillance programme had been described as the ‘pathfinder’ for GLASS but is at a considerable advantage with the development of robust molecular detection methods, notably the roll-out of GeneXpert^®^, a PCR-based technology that can be performed directly on primary TB specimens without an intermediate culture step.^18^ Molecular surveillance for drug resistance in other bacteria remains some way off but should be made a high priority in order to simplify surveillance in LMICs.

Assessing the representativeness of AMR surveillance data presents a particular challenge. This will be affected by the geographical location and number of sentinel sites, the number and characteristics of individuals sampled, prior treatment history, the incidence of the target pathogen and the methods of detection. WHO/IUATLD has developed its surveillance methodology to the point where it uses survey data to estimate MDR-TB incidence worldwide but this is exceptional for the global programmes. The global report on early warning indicators of HIV drug resistance states that data from most countries cannot be considered as representative due to the way in which the clinics sampled were selected.^11^ In malaria therapeutic efficacy studies in high-transmission settings, children less than five years of age are studied since they have the lowest levels of acquired immunity to malaria to give a ‘worst-case scenario’ depiction of drug efficacy. AMR surveillance for the most commonly encountered bacteria, as it has been practised to date, presents more problems than for other pathogens because of the lack of agreed case definitions and standardized sampling methods. An analysis comparing trends in *Escherichia coli* resistance from 1997 to 2001 reported by the global Meropenem Yearly Susceptible Test Information Collection (MYSTIC) and SENTRY pharma networks showed that, despite collecting isolates from similar geographical areas, estimates of non-susceptibility from MYSTIC were consistently higher than those from SENTRY. However, further analysis revealed this was due to a higher proportion of isolates from patients in ICUs in MYSTIC.^19^

AMR surveillance in animals is still in its infancy, with the exception of foodborne infections, but some strategies have been piloted in LMICs under the guidance of the WHO Advisory Group on Integrated Surveillance of Antimicrobial Resistance (AGISAR). The challenges are great, e.g. progress towards standardizing AST breakpoints in veterinary microbiology is far behind that made in humans.

Other networks deserving of a mention that were not included in this analysis are two Digital Disease Detection networks, ProMed and HealthMap, which publish sporadic AMR reports and have an advantage over other networks for the rapidity with which they disseminate information. There is potential for overlap between the activities of networks for AMR detection, foodborne infections and emerging disease detection.

The main limitation of our approach is that the heterogeneity of the data meant meta-analysis was not possible. There are no recognized standards for the composition and activities of AMR surveillance networks. Impacts and challenges of the networks were reported infrequently and our assessment is reliant on published information, which may be more likely to report challenges. In addition, our search was only performed in English with a supplementary search in Spanish to obtain more information about the Latin-American networks.

A successful AMR surveillance network should generate up-to-date comparable, representative, high-quality data on pathogens of concern from the target population(s). It should be able to detect and track unexpected events including outbreaks in real time, have rapid, effective mechanisms for communication and reporting, and have a responsible data-sharing policy. A network needs strong leadership and coordination, and it should influence guidelines and policy and ultimately impact on human and animal health. Very few networks were instigated to specifically monitor intervention programmes, e.g. the International Nosocomial Infection Control Consortium. Linking surveillance activity to interventions to combat drug resistance has the potential to increase their impact.

Pharma networks produce high-quality data, but they may not be representative and these networks do not usually support laboratory capacity building in LMICs or influence policy and guidelines. Purely academic networks also produce high-quality data; they often target a clinical or policy question, but they too have limited influence on policy and their sustainability is reliant on external funding. Most of the networks are slow to report their findings and do not give unrestricted access to their data. The experience of the larger global programmes for AMR surveillance in TB, malaria and HIV suggests that options for more active surveillance may need to be considered in order to gather comparable useful data from low-income countries before reliable case-based surveillance can be established.

Maintaining an up-to-date registry of networks would promote a more coordinated approach to surveillance, reduce duplication of efforts, optimize funding investment and improve sustainability.

## Supplementary Material

Supplementary Data
