# Supplementary material for: An inventory of supranational antimicrobial resistance surveillance networks involving low- and middle-income countries since 2000
Source: J Antimicrob Chemother. 2018 Mar 5;73(7):1737–49. doi: 10.1093/jac/dky026 (PMC6005144; doi:10.1093/jac/dky026)
Supplement: Supplementary Data [file dky026_supplementary-data.docx]

**Supplementary data**

**1. Search Strategy: Networks involved in the surveillance of antimicrobial drug resistance in low-middle income countries.** Search performed on 05/05/16

**Database: Embase <1974 to 2016 May 04>**

Search Strategy:

--------------------------------------------------------------------------------

1 antibiotic resistance/ (126544)

2 drug resistance/ or exp antibiotic resistance/ or exp antifungal resistance/ or exp antimalarial drug resistance/ or exp antiviral resistance/ or exp cross resistance/ or exp multidrug resistance/ or exp penicillin resistance/ or exp pesticide resistance/ (239457)

3 exp drug resistant tuberculosis/ (5477)

4 ((antibiotic* or anti-biotic*) adj3 resistan*).ti,ab. (42435)

5 (bacterial adj3 resistan*).ti,ab. (11939)

6 ((anti-fungal* or antifungal*) adj3 susceptib*).ti,ab. (3080)

7 ((anti-fungal* or antifungal*) adj3 surveillan*).ti,ab. (72)

8 ((anti-fungal* or antifungal*) adj3 resistan*).ti,ab. (1969)

9 (HIV* adj3 resistan*).ti,ab. (7753)

10 ((antiretroviral* or anti-retroviral*) adj3 resistan*).ti,ab. (1780)

11 ((antimalarial* or anti-malarial*) adj3 resistan*).ti,ab. (1191)

12 ((anti-tuberculosis or antituberculosis) adj3 resistan*).ti,ab. (571)

13 "MDR tuberculosis".ti,ab. (345)

14 (((multidrug* or multi-drug*) and tuberculosis) adj3 resistan*).ti,ab. (7303)

15 ((antimicrobial* or anti-microbial*) adj3 resistan*).ti,ab. (18931)

16 ((antimicrobial* or anti-microbial*) adj3 surveillan*).ti,ab. (1700)

17 ((antimicrobial* or anti-microbial*) adj3 susceptib*).ti,ab. (14093)

18 AMR.ti,ab. (3179)

19 ((antibacterial* or anti-bacterial*) adj3 resistan*).ti,ab. (1444)

20 OR/ 1-19 (281488)

21 surveillan*.ti,ab. (163513)

22 exp drug surveillance program/ (21554)

23 exp prevalence/ (497896)

24 exp health survey/ (183834)

25 OR/ 21-24 (802958)

26 developing country/ (83513)

27 exp "Africa south of the Sahara"/ or exp Africa/ (249775)

28 exp Asia/ (758868)

29 exp South America/ (143140)

30 exp "South and Central America"/ (167411)

31 (Africa or Asia or South America or Latin America or Central America).tw. (158960)

32 (American Samoa$ or Beliz$ or Botswana$ or Brazil$ or Bulgaria$ or Comoro$ or Costa Rica$ or Croatia$ or Dominica$ or Equatorial Guinea$ or Gabon$ or Grenada$ or Kazakh$).tw. (128433)

33 (Leban$ or Libya$ or Lithuania$ or Malaysia$ or Mauriti$ or Mexic$ or Micronesia$ or Montenegr$ or Palau$ or Panama$ or Romania$).tw. (97345)

34 (Seychelles$ or South Africa$ or Saint Lucia$ or "Saint Vincent and the Grenadines" or Turk$).tw. (98504)

35 (Yugoslavia$ or Guinea$ or Libia$ or Mayotte or Northern Mariana Island$ or Russian Federation or Samoa$ or Serbia$ or Slovak Republic$).tw. (126207)

36 (St Lucia$ or "St Vincent and the Grenadines").tw. (295)

37 (Albania$ or Algeria$ or Angol$ or Armenia$ or Azerbaijan$ or Belarus$ or Bhutan$ or Bolivia$ or "Bosnia and Herzegovina" or Bosnian$).tw. (18448)

38 (Cameroon$ or China or Chinese or Colombia$ or Congo$ or Cuba$ or Djibouti$ or Dominican Republic$ or Ecuador$ or Egypt$ or El Salvador$ or Fiji$).tw. (345372)

39 ("Georgia (Republic)" or Goergian$ or Guam$ or Guatemal$ or Guyana$ or Hondur$ or Indian Ocean Island$ or Indonesia$ or Iran$ or Iraq$ or Jamaica$ or Jordan$ or Lesotho).tw. (77648)

40 ("Macedonia (Republic)" or Marshall Island$ or Micronesia$ or Middle East$ or Moldova$ or Morocc$ or Namibia$ or Nicaragua$ or Paraguay$ or Peru$ or Philippin$).tw. (46055)

41 (Samoa$ or Sri Lanka$ or Suriname$ or Swaziland$ or Syria$ or Thai$ or Tonga$ or Tunisia$ or Turkmen$ or Ukrain$ or Vanuatu).tw. (72724)

42 (Bosnia$ or Cape Verd$ or Gaza or Georgia$ or Kiribati$ or Macedonia$ or Maldives or Marshall Island$ or Palestin$ or Syrian Arab Republic$ or West Bank).tw. (17626)

43 (Afghan$ or Bangladesh$ or Benin$ or Burkina Faso$ or Burundi$ or Cambodia$ or Central African Republic$ or Chad$ or Comoros or "Democratic Republic of the Congo").tw. (36779)

44 (Cote d'Ivoire or Eritrea$ or Ethiopia$ or Gambia$ or Ghana$ or Guinea$ or Guinea-Bissau or Haiti$ or India$ or Kenya$ or Korea$ or Kyrgyz$ or Laos or Laot$ or Liberia$).tw. (391775)

45 (Madagascar or Malagasy or Malawi$ or Mali$ or Mauritania$ or Melanesia$ or Mongolia$ or Mozambi$ or Myanmar or Nepal$ or Niger$ or Nigeria$).tw. (704503)

46 (Pakistan$ or Papua New Guinea$ or Rwanda$ or Senegal$ or Sierra Leone$ or Somalia$ or Sudan$ or Tajikistan$ or Tanzania$ or East Timor$ or Togo$).tw. (55448)

47 (Uganda$ or Uzbek$ or Viet Nam$ or Yemen$ or Zambia$ or Zimbabw$).tw. (38488)

48 (Burm$ or Congo$ or Lao or North Korea$ or Solomon Island$ or Sao Tome or Timor$ or Viet Nam).tw. (21555)

49 ((developing or less$ developed or third world or under developed or middle income or low income or underserved or under served or deprived or poor$) adj (count$ or nation? or state? or population?)).tw. (78367)

50 (lmic or lmics).tw. (1460)

51 OR/ 26-50 (2572745)

52 20 and 25 and 51 (10122)

53 52 (10122)

54 limit 53 to yr="2000 -Current" (9525)

**Database: Global Health <1973 to 2016 Week 16>**

Search Strategy:

--------------------------------------------------------------------------------

1 exp drug resistance/ (78375)

2 drug resistance/ or exp antibiotic resistance/ or exp antifungal resistance/ or exp antimalarial drug resistance/ or exp antiviral resistance/ or exp cross resistance/ or exp multidrug resistance/ or exp penicillin resistance/ or exp pesticide resistance/ (72384)

3 ((antibiotic* or anti-biotic*) adj3 resistan*).ti,ab. (17385)

4 (bacterial adj3 resistan*).ti,ab. (3476)

5 ((anti-fungal* or antifungal*) adj3 susceptib*).ti,ab. (2153)

6 ((anti-fungal* or antifungal*) adj3 surveillan*).ti,ab. (49)

7 ((anti-fungal* or antifungal*) adj3 resistan*).ti,ab. (1240)

8 (HIV* adj3 resistan*).ti,ab. (3265)

9 ((antiretroviral* or anti-retroviral*) adj3 resistan*).ti,ab. (1007)

10 ((antimalarial* or anti-malarial*) adj3 resistan*).ti,ab. (844)

11 ((anti-tuberculosis or antituberculosis) adj3 resistan*).ti,ab. (337)

12 "MDR tuberculosis".ti,ab. (200)

13 (((multidrug* or multi-drug*) and tuberculosis) adj3 resistan*).ti,ab. (3858)

14 ((antimicrobial* or anti-microbial*) adj3 resistan*).ti,ab. (10807)

15 ((antimicrobial* or anti-microbial*) adj3 surveillan*).ti,ab. (962)

16 ((antimicrobial* or anti-microbial*) adj3 susceptib*).ti,ab. (8418)

17 AMR.ti,ab. (317)

18 ((antibacterial* or anti-bacterial*) adj3 resistan*).ti,ab. (619)

19 OR/ 1-18 (90507)

20 surveillan*.ti,ab. (56704)

21 (monitoring or surveillance).sh. (36622)

22 20 OR 21 (76845)

23 exp Developing Countries/ (625454)

24 exp "Africa south of the Sahara"/ or exp Africa/ (154943)

25 exp Asia/ (427221)

26 exp South America/ (94870)

27 (South and Central America).mp. [mp=abstract, title, original title, broad terms, heading words, identifiers, cabicodes] (2568)

28 exp Central America/ (7677)

29 (Africa or Asia or South America or Latin America or Central America).tw. (703297)

30 (American Samoa$ or Beliz$ or Botswana$ or Brazil$ or Bulgaria$ or Comoro$ or Costa Rica$ or Croatia$ or Dominica$ or Equatorial Guinea$ or Gabon$ or Grenada$ or Kazakh$).tw. (84768)

31 (Leban$ or Libya$ or Lithuania$ or Malaysia$ or Mauriti$ or Mexic$ or Micronesia$ or Montenegr$ or Palau$ or Panama$ or Romania$).tw. (46386)

32 (Seychelles$ or South Africa$ or Saint Lucia$ or "Saint Vincent and the Grenadines" or Turk$).tw. (50066)

33 (Yugoslavia$ or Guinea$ or Libia$ or Mayotte or Northern Mariana Island$ or Russian Federation or Samoa$ or Serbia$ or Slovak Republic$).tw. (28623)

34 (St Lucia$ or "St Vincent and the Grenadines").tw. (308)

35 (Albania$ or Algeria$ or Angol$ or Armenia$ or Azerbaijan$ or Belarus$ or Bhutan$ or Bolivia$ or "Bosnia and Herzegovina" or Bosnian$).tw. (9286)

36 (Cameroon$ or China or Chinese or Colombia$ or Congo$ or Cuba$ or Djibouti$ or Dominican Republic$ or Ecuador$ or Egypt$ or El Salvador$ or Fiji$).tw. (195257)

37 ("Georgia (Republic)" or Goergian$ or Guam$ or Guatemal$ or Guyana$ or Hondur$ or Indian Ocean Island$ or Indonesia$ or Iran$ or Iraq$ or Jamaica$ or Jordan$ or Lesotho).tw. (53920)

38 ("Macedonia (Republic)" or Marshall Island$ or Micronesia$ or Middle East$ or Moldova$ or Morocc$ or Namibia$ or Nicaragua$ or Paraguay$ or Peru$ or Philippin$).tw. (88185)

39 (Samoa$ or Sri Lanka$ or Suriname$ or Swaziland$ or Syria$ or Thai$ or Tonga$ or Tunisia$ or Turkmen$ or Ukrain$ or Vanuatu).tw. (34452)

40 (Bosnia$ or Cape Verd$ or Gaza or Georgia$ or Kiribati$ or Macedonia$ or Maldives or Marshall Island$ or Palestin$ or Syrian Arab Republic$ or West Bank).tw. (8159)

41 (Afghan$ or Bangladesh$ or Benin$ or Burkina Faso$ or Burundi$ or Cambodia$ or Central African Republic$ or Chad$ or Comoros or "Democratic Republic of the Congo").tw. (22192)

42 (Cote d'Ivoire or Eritrea$ or Ethiopia$ or Gambia$ or Ghana$ or Guinea$ or Guinea-Bissau or Haiti$ or India$ or Kenya$ or Korea$ or Kyrgyz$ or Laos or Laot$ or Liberia$).tw. (176846)

43 (Madagascar or Malagasy or Malawi$ or Mali$ or Mauritania$ or Melanesia$ or Mongolia$ or Mozambi$ or Myanmar or Nepal$ or Niger$ or Nigeria$).tw. (82145)

44 (Pakistan$ or Papua New Guinea$ or Rwanda$ or Senegal$ or Sierra Leone$ or Somalia$ or Sudan$ or Tajikistan$ or Tanzania$ or East Timor$ or Togo$).tw. (38106)

45 (Uganda$ or Uzbek$ or Viet Nam$ or Yemen$ or Zambia$ or Zimbabw$).tw. (23056)

46 (Burm$ or Congo$ or Lao or North Korea$ or Solomon Island$ or Sao Tome or Timor$ or Viet Nam).tw. (18038)

47 ((developing or less$ developed or third world or under developed or middle income or low income or underserved or under served or deprived or poor$) adj (count$ or nation? or state? or population?)).tw. (636179)

48 (lmic or lmics).tw. (648)

49 OR/ 23-48 (875208)

50 19 and 22 and 49 (4378)

51 limit 50 to yr="2000 -Current" (4125)

PubMed – search date 05/05/16

(((((((((((((((((((((((((lmic[Text Word] OR lmics[Text Word]))) OR (((developing[Text Word] OR less$ developed[Text Word] OR third world[Text Word] OR under developed[Text Word] OR middle income[Text Word] OR low income[Text Word] OR underserved[Text Word] OR under served[Text Word] OR deprived[Text Word] OR poor$)[Text Word] AND (count$[Text Word] OR nation?[Text Word] OR state?[Text Word] OR population?)[Text Word]))) OR ((Burm$[Text Word] OR Congo$[Text Word] OR Lao[Text Word] OR North Korea$[Text Word] OR Solomon Island$[Text Word] OR Sao Tome[Text Word] OR Timor$[Text Word] OR Viet Nam[Text Word]))) OR ((Uganda$[Text Word] OR Uzbek$[Text Word] OR Viet Nam$[Text Word] OR Yemen$[Text Word] OR Zambia$[Text Word] OR Zimbabw$[Text Word]))) OR ((Pakistan$[Text Word] OR Papua New Guinea$[Text Word] OR Rwanda$[Text Word] OR Senegal$[Text Word] OR Sierra Leone$[Text Word] OR Somalia$[Text Word] OR Sudan$[Text Word] OR Tajikistan$[Text Word] OR Tanzania$[Text Word] OR East Timor$[Text Word] OR Togo$[Text Word]))) OR ((Madagascar[Text Word] OR Malagasy[Text Word] OR Malawi$[Text Word] OR Mali$[Text Word] OR Mauritania$[Text Word] OR Melanesia$[Text Word] OR Mongolia$[Text Word] OR Mozambi$[Text Word] OR Myanmar[Text Word] OR Nepal$[Text Word] OR Niger$[Text Word] OR Nigeria$[Text Word]))) OR ((Cote d'Ivoire[Text Word] OR Eritrea$[Text Word] OR Ethiopia$[Text Word] OR Gambia$[Text Word] OR Ghana$[Text Word] OR Guinea$[Text Word] OR Guinea-Bissau[Text Word] OR Haiti$[Text Word] OR India$[Text Word] OR Kenya$[Text Word] OR Korea$[Text Word] OR Kyrgyz$[Text Word] OR Laos[Text Word] OR Laot$[Text Word] OR Liberia$[Text Word]))) OR ((Afghan$[Text Word] OR Bangladesh$[Text Word] OR Benin$[Text Word] OR Burkina Faso$[Text Word] OR Burundi$[Text Word] OR Cambodia$[Text Word] OR Central African Republic$[Text Word] OR Chad$[Text Word] OR Comoros[Text Word] OR "Democratic Republic of the Congo"[Text Word]))) OR ((Bosnia$[Text Word] OR Cape Verd$[Text Word] OR Gaza[Text Word] OR Georgia$[Text Word] OR Kiribati$[Text Word] OR Macedonia$[Text Word] OR Maldives[Text Word] OR Marshall Island$[Text Word] OR Palestin$[Text Word] OR Syrian Arab Republic$[Text Word] OR West Bank[Text Word]))) OR ((Samoa$[Text Word] OR Sri Lanka$[Text Word] OR Suriname$[Text Word] OR Swaziland$[Text Word] OR Syria$[Text Word] OR Thai$[Text Word] OR Tonga$[Text Word] OR Tunisia$[Text Word] OR Turkmen$[Text Word] OR Ukrain$[Text Word] OR Vanuatu[Text Word]))) OR (("Macedonia (Republic)"[Text Word] OR Marshall Island$[Text Word] OR Micronesia$[Text Word] OR Middle East$[Text Word] OR Moldova$[Text Word] OR Morocc$[Text Word] OR Namibia$[Text Word] OR Nicaragua$[Text Word] OR Paraguay$[Text Word] OR Peru$[Text Word] OR Philippin$[Text Word]))) OR (("Georgia (Republic)"[Text Word] OR Goergian$[Text Word] OR Guam$[Text Word] OR Guatemal$[Text Word] OR Guyana$[Text Word] OR Hondur$[Text Word] OR Indian Ocean Island$[Text Word] OR Indonesia$[Text Word] OR Iran$[Text Word] OR Iraq$[Text Word] OR Jamaica$[Text Word] OR Jordan$[Text Word] OR Lesotho[Text Word]))) OR ((Cameroon$[Text Word] OR China[Text Word] OR Chinese[Text Word] OR Colombia$[Text Word] OR Congo$[Text Word] OR Cuba$[Text Word] OR Djibouti$[Text Word] OR Dominican Republic$[Text Word] OR Ecuador$[Text Word] OR Egypt$[Text Word] OR El Salvador$[Text Word] OR Fiji$[Text Word]))) OR ((Albania$[Text Word] OR Algeria$[Text Word] OR Angol$[Text Word] OR Armenia$[Text Word] OR Azerbaijan$[Text Word] OR Belarus$[Text Word] OR Bhutan$[Text Word] OR Bolivia$[Text Word] OR "Bosnia[Text Word] AND Herzegovina"[Text Word] OR Bosnian$[Text Word]))) OR ((St Lucia$[Text Word] OR "St Vincent[Text Word] AND the Grenadines"[Text Word]))) OR ((Yugoslavia$[Text Word] OR Guinea$[Text Word] OR Libia$[Text Word] OR Mayotte[Text Word] OR Northern Mariana Island$[Text Word] OR Russian Federation[Text Word] OR Samoa$[Text Word] OR Serbia$[Text Word] OR Slovak Republic$[Text Word]))) OR ((Seychelles$[Text Word] OR South Africa$[Text Word] OR Saint Lucia$[Text Word] OR "Saint Vincent[Text Word] AND the Grenadines"[Text Word] OR Turk$[Text Word]))) OR ((Leban$[Text Word] OR Libya$[Text Word] OR Lithuania$[Text Word] OR Malaysia$[Text Word] OR Mauriti$[Text Word] OR Mexic$[Text Word] OR Micronesia$[Text Word] OR Montenegr$[Text Word] OR Palau$[Text Word] OR Panama$[Text Word] OR Romania$[Text Word]))) OR ((American Samoa$[Text Word] OR Beliz$[Text Word] OR Botswana$[Text Word] OR Brazil$[Text Word] OR Bulgaria$[Text Word] OR Comoro$[Text Word] OR Costa Rica$[Text Word] OR Croatia$[Text Word] OR Dominica$[Text Word] OR Equatorial Guinea$[Text Word] OR Gabon$[Text Word] OR Grenada$[Text Word] OR Kazakh$[Text Word]))) OR ((Africa[Text Word] OR Asia[Text Word] OR South America[Text Word] OR Latin America[Text Word] OR Central America[Text Word]))) OR (((((("Developing Countries"[Mesh]) OR ( "Africa"[Mesh] OR "Africa South of the Sahara"[Mesh] )) OR "Asia"[Mesh]) OR "South America"[Mesh]) OR "Latin America"[Mesh]) OR "Central America"[Mesh]))) AND ((surveillan*[Title/Abstract]) OR ((("Prevalence"[Mesh]) OR "Health Surveys"[Mesh]) OR "Guideline Adherence"[Mesh]))) AND ((((((((((((((((("Tuberculosis, Multidrug-Resistant"[Mesh]) OR ("Drug Resistance, Microbial"[Mesh]) OR "Drug Resistance, Multiple"[Mesh])) OR ((resistan*[Title/Abstract]) AND ((anti-biotic*[Title/Abstract] OR antibiotic*[Title/Abstract])))) OR ((resistan*[Title/Abstract]) AND ((bacterial[Title/Abstract] OR anti-bacterial*[Title/Abstract] OR antibacterial*[Title/Abstract])))) OR ((resistan*[Title/Abstract]) AND ((anti-fungal*[Title/Abstract] OR antifungal*[Title/Abstract])))) OR ((resistan*[Title/Abstract]) AND HIV*[Title/Abstract])) OR ((surveillan*[Title/Abstract]) AND ((anti-fungal*[Title/Abstract] OR antifungal*[Title/Abstract])))) OR ((((anti-fungal*[Title/Abstract] OR antifungal*[Title/Abstract]))) AND susceptib*[Title/Abstract])) OR ((resistan*[Title/Abstract]) AND ((anti-retroviral*[Title/Abstract] OR antiretroviral*[Title/Abstract])))) OR ((resistan*[Title/Abstract]) AND ((antimalarial*[Title/Abstract] OR anti-malarial*[Title/Abstract])))) OR ((resistan*[Title/Abstract]) AND ((anti-tuberculosis[Title/Abstract] OR antituberculosis[Title/Abstract])))) OR (((resistan*[Title/Abstract]) AND (multidrug*[Title/Abstract] OR multi-drug*)[Title/Abstract]) AND tuberculosis[Title/Abstract])) OR ((resistan*[Title/Abstract]) AND ((anti-microbial*[Title/Abstract] OR antimicrobial*[Title/Abstract])))) OR ((((anti-microbial*[Title/Abstract] OR antimicrobial*[Title/Abstract]))) AND surveillan*[Title/Abstract])) OR ((susceptib*[Title/Abstract]) AND ((anti-microbial*[Title/Abstract] OR antimicrobial*[Title/Abstract])))) OR MDR tuberculosis[Title/Abstract]) OR AMR[Title/Abstract])

Filters activated: Publication date from 2000/01/01 to 2016/12/31.

= 7102

Search repeated on 14^th^ August 2017 (Pubmed publication date from 5/5/2016 to 14/08/2017; Embase and Global Health Databases publication date from 2016-14/08/2017).
